# Helium’s placement in the Periodic Table from a crystal structure viewpoint

**DOI:** 10.1107/S2052252520007769

**Published:** 2020-06-13

**Authors:** Mikhail Kurushkin

**Affiliations:** a Chemistry Education Research and Practice Laboratory, SCAMT Institute, ITMO University, 9 Lomonosova Str., Saint Petersburg, Saint Petersburg 191002, Russian Federation

**Keywords:** Periodic Table, helium, crystal structure

## Abstract

The present paper indicates that helium has more in common with the alkaline earths than is often considered. Not only does it share an analogous electron configuration but it also has an analogous crystal structure to that of beryllium and magnesium which is, in contrast, characteristic of the bulk material rather than of isolated atoms.

By 2019, one hundred and fifty years after Dmitry Mendeleev published the first successful version of the Periodic Table of the chemical elements, there was still no universal agreement regarding what a chemical element is. A notable indication of the ongoing ambiguity is IUPAC’s *Gold Book*, which allows two different versions of the term ‘chemical element’: (1) a species of atoms – all atoms with the same number of protons in the atomic nucleus; (2) a pure chemical substance composed of atoms with the same number of protons in the atomic nucleus. W. H. E. Schwarz in his 2007 paper (Schwarz, 2007 ▸) argued that there are in fact three different definitions of a chemical element usually encountered: (1) a basic chemical element; (2) a metallurgical element or simple material; (3) an astrophysical spectroscopic element or elemental atom. W. B. Jensen suggested a definition (Jensen, 1998 ▸) which focuses on atomic nuclei rather on neutral atoms: (1) a class of nuclei, all of which have the same atomic number. The one question that permanently accompanies the definition of the chemical element is the representation of the Periodic Table itself.

The most common version of the revered icon of chemistry is the IUPAC Periodic Table of the Elements. Whichever representation of the periodic system is argued to be the optimal one (Leigh, 2009 ▸; Scerri, 2009 ▸), consistency of representation is the criterion that has to be met.

The IUPAC Periodic Table has four blocks of chemical elements: the *s*-, *p*-, *d*- and *f*-blocks, hence its whole body is based on electron configurations. One hundred and seventeen of the known elements fit into those blocks; however, there is only one element, helium, placed on top of the *p*-block as it is a noble gas. Hence, the representation becomes inconsistent overall because the Periodic Table simultaneously adopts two different definitions of the chemical element. According to the *Gold Book*, we then have one hundred and seventeen species of atoms and one pure chemical substance. Thus, such a placement of helium transforms the Periodic Table of Chemical Elements into the Periodic Table of Pure Substances.

Switching to the Periodic Table of Pure Substances would inevitably make us consider two further questions: (1) states of aggregation; (2) allotropes. As E. R. Scerri fairly states, in case of the Periodic Table of Pure Substances, ‘one would probably not consider grouping together fluorine and chlorine, two green–yellow gases, along with a brown liquid bromine and a violet–black solid such as iodine’ (Scerri, 2005 ▸). Numerous similar examples can be provided. Which temperature and pressure do we choose for the representation? Which allotrope(s) do we prefer? As a matter of convention, we can choose standard temperature and pressure. But what about allotropes, the physical forms of chemical elements? We would need to either choose one of the allotropes or incorporate them all in one place, which does not seem rational. It can be seen that the choice of a Periodic Table of Pure Substances over the Periodic Table of Chemical Elements would probably cause an overcomplicated representation.

We now return to helium, the noble gas. In the majority of the common versions of the Periodic Table one can always find elements classified as solids, liquids and gases. However, for the sake of the observation in this paper, it is suggested that all the pure substances are considered in their solid state so that van der Waals forces become pronounced. The question will then be, would solid helium above solid beryllium be regarded as equally irregular as the case of gaseous helium above solid beryllium? To be more specific, in the solid state, would we still support the idea of putting helium above neon *because* they are both noble gases? It is well known that solid helium has been obtained and characterized with its crystal structure being hexagonal close-packed (Donohue, 1959 ▸).

Next, it might be considered surprising that solid helium, beryllium and magnesium all have the same crystal structure (Sluiter, 2007 ▸; Luo *et al.*, 2012 ▸), which is hexagonal close-packed (h.c.p.); while neon, argon, krypton and xenon all have a face-centred cubic (f.c.c.) crystal structure (Sonnenblick *et al.*, 1982 ▸; Moyano *et al.*, 2007 ▸) (Fig. 1 ▸).

Most recent publications dedicated to the placement of helium clearly demonstrate that the topic has never been more relevant (Labarca & Srivaths, 2016 ▸, 2017 ▸; Cvetković & Petruševski, 2017 ▸; Grochala, 2018 ▸). In his most recent essay, Scerri highlights the two most common opposing views regarding the placement of helium: (1) it should be grouped with the rest of the noble gases; (2) it should be grouped with the alkaline earths because of an *s* ^2^ configuration, but that means the reduction of chemistry to quantum mechanics (Scerri, 2019*b* ▸). In another 2019 paper, however, Scerri (2019*a* ▸) theorizes that a ‘deep dive into quantum mechanics’ might actually facilitate our understanding of the fundamental aspects of the periodic system.

For instance, the latter is usually the argument of Left-step Periodic Table supporters (Scerri, 2012*b* ▸; Kurushkin, 2017 ▸). However, such an approach has not found recognition among chemists due to the very low reactivity of helium (Scerri, 2012*a* ▸), a view that might be reversed in the near future as new exotic stable compounds of helium (Na_2_He) are being discovered thanks to the *ab initio* evolutionary algorithm USPEX (Dong *et al.*, 2017 ▸). Furthermore, unusual helium-bearing compounds (FeHe, FeHe_2_, FeO_2_He), stable under extreme conditions, have also been reported recently (Zhang *et al.*, 2018 ▸; Monserrat *et al.*, 2018 ▸).

Helium turns out to have more in common with the alkaline earths than is often considered. Not only does it share an analogous electron configuration, but it also has an analogous crystal structure to that of beryllium and magnesium which is, in contrast, characteristic of the bulk material rather than of isolated atoms. The latter, importantly, means that the placement of helium above beryllium is not solely a reduction to quantum mechanics. Last but not least, recent *ab initio* calculations (Bakai *et al.*, 2011 ▸) have shown substantial He–Be bonding in h.c.p.-beryllium.

## Figures and Tables

**Figure 1:**
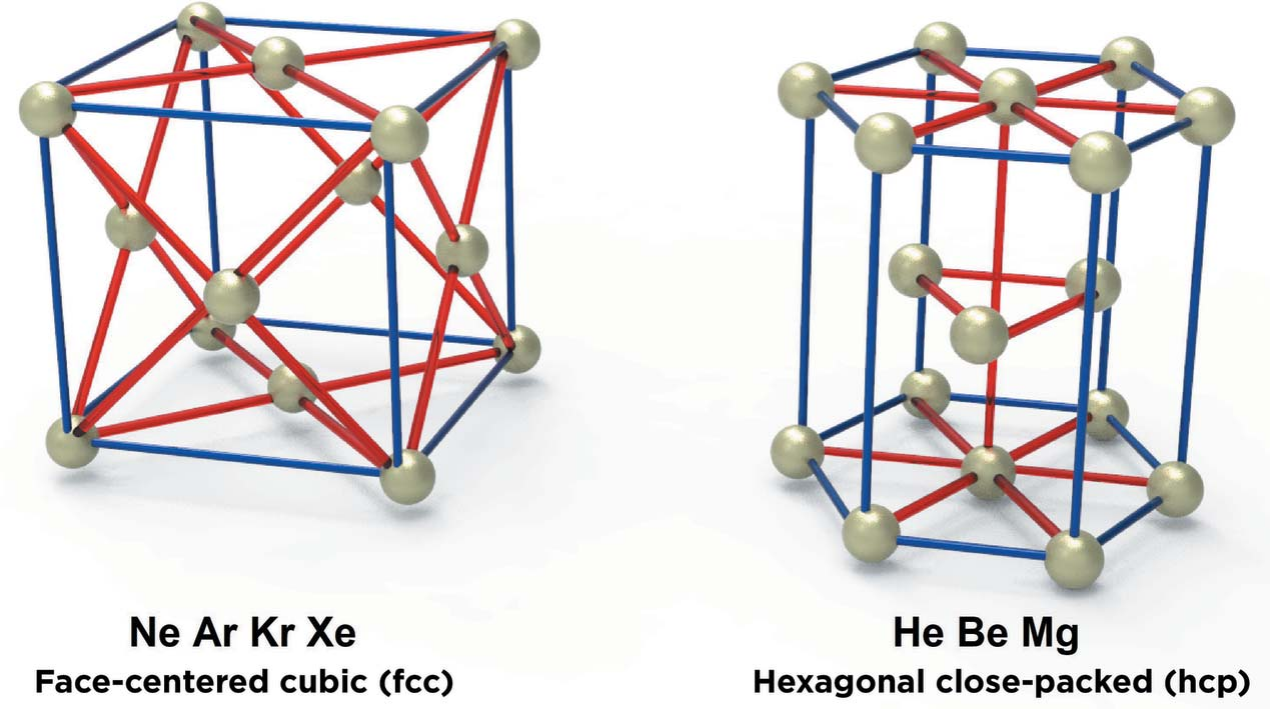
Face-centred cubic (neon, argon, krypton and xenon) and hexagonal close-packed (helium, beryllium and magnesium) crystal structures.
